# Imaging of the aortic root on high-pitch non-gated and ECG-gated CT: awareness is the key!

**DOI:** 10.1186/s13244-020-00855-w

**Published:** 2020-03-20

**Authors:** Prashant Nagpal, Mukta D. Agrawal, Sachin S. Saboo, Sandeep Hedgire, Sarv Priya, Michael L. Steigner

**Affiliations:** 1 Department of Radiology, University of Iowa Hospitals and Clinics, Iowa City, IA USA; 2 Department of Radiology, Non-invasive Cardiovascular Imaging, Brigham and Women Hospital, Harvard Medical School, Boston, MA USA; 3 Department of Radiology, Oklahoma University Health Sciences Center, Oklahoma City, OK USA; 4 Department of Radiology, University of Texas Health Center, San Antonio, TX USA; 5 Department of Radiology, Cardiovascular Imaging, Massachusetts General Hospital, Harvard Medical School, Boston, MA USA

**Keywords:** High-pitch CT, Aortic root, CT angiography, Aneurysm, Aortic valve

## Abstract

The aortic pathologies are well recognized on imaging. However, conventionally cardiac and proximal aortic abnormalities were only seen on dedicated cardiac or aortic studies due to need for ECG gating. Advances in CT technology have allowed motionless imaging of the chest and abdomen, leading to an increased visualization of cardiac and aortic root diseases on non-ECG-gated imaging. The advances are mostly driven by high pitch due to faster gantry rotation and table speed. The high-pitch scans are being increasingly used for variety of clinical indications because the images are free of motion artifact (both breathing and pulsation) as well as decreased radiation dose. Recognition of aortic root pathologies may be challenging due to lack of familiarity of radiologists with disease spectrum and their imaging appearance. It is important to recognize some of these conditions as early diagnosis and intervention is key to improving prognosis. We present a comprehensive review of proximal aortic anatomy, pathologies commonly seen at the aortic root, and their imaging appearances to familiarize radiologists with the diseases of this location.

## Key points

- Advances in CT technology have allowed acquisition of imaging without cardiac pulsation artifact even without ECG gating.
- Such protocols are being increasingly used for non-aortic indications due to better image quality and less patient radiation dose.
- Aortic root and ascending aortic pathologies can be incidentally seen in patients getting a CT for non-aortic indications.
- Knowledge of the pathologies specific to this part of the aorta and their imaging appearance is useful for diagnosis and early treatment.

## Background

The diseases of the aorta are life threatening and are being increasingly diagnosed due to increased use and availability of imaging [1]. Epidemiologically, the incidence of the most life-threatening aortic disorder, aortic dissection (AD), has increased over time, which could be due to better detection with improved imaging or increased longevity [1, 2]. More importantly, the advances in imaging technology have facilitated the assessment of cardiac structures and proximal aorta on “routine” non-electrocardiogram (ECG)-gated chest CTs. These advances are mostly driven by faster gantry rotations, faster table speed, and sometimes by the use of more than one X-ray source, allowing high-pitch exams. Such high-pitch CT scans have allowed aortic evaluation even without ECG gating [3–5]. The high-pitch scans are being increasingly used for non-aortic clinical indications because of lack of motion (both breathing and pulsation) artifacts as well as decreased radiation dose [3].

Studies comparing the high-pitch and routine scanning for pulmonary embolism CT have shown improved image quality, decreased artifacts, and decreased radiation dose with high-pitch imaging [6, 7]. This improvement in image quality is associated with better delineation of cardiovascular structures. A clinical trial, Cardiac Pathologies in standard chest CT (CaPaCT) study [8], and other studies [9, 10] have highlighted that there is increased detection of cardiovascular incidentals on routine non-ECG-gated chest CTs. In a study, Verdini et al. [10] showed that the sensitivity for detection of cardiovascular incidentals correlates with the reader experience with increased sensitivity if the reader has dedicated cardiac training. In a single-center retrospective study, Seechi et al. [9] showed that incidental cardiovascular findings were seen in > 50% of retrospectively evaluated cases (124/237), out of which aortic “incidental” finding was presented in 34.9% cases (80/229). Significantly, they also remarked that cardiovascular “incidentals” were not mentioned in nearly 70% of cases. While these “incidental” findings without clinical context have a concern of overdiagnosis and increase in patient management burden, delayed diagnosis of a critical finding (especially proximal aortic finding) may cause significant morbidity and mortality.

The limitations to the diagnosis of the aortic root and ascending aortic diseases include lack of familiarity of the anatomy and the imaging appearance of specific conditions among non-cardiothoracic radiologists. Moreover, improved visualization of the proximal aorta on scans obtained on scanners with faster gantry rotation and their interpretation by radiologists that may not be familiar with these diseases has added to this challenge. Although technological advances have happened in other aortic imaging modalities like magnetic resonance angiography, and trans-esophageal echocardiography [2], incidentally seen aortic pathologies are mostly described with CT. Hence, we aim to present a review of proximal aortic anatomy, various disorders that affect the aortic root, and their imaging appearances to familiarize radiologists with the diseases of this location.

## Main text

### Dedicated CT imaging for aortic root evaluation

**Fig. 1:**
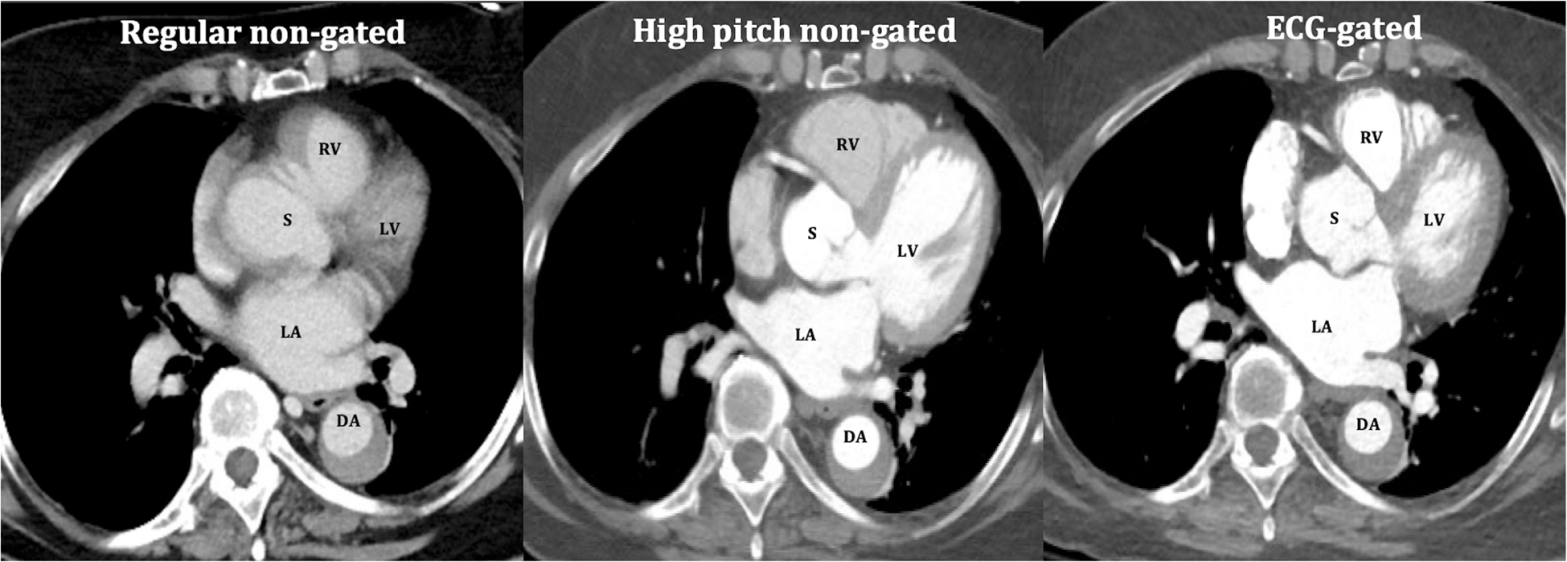
A 67-year-old man with medically managed type B aortic dissection and metastatic colon cancer. Conventional non-ECG-gated CT chest image, high-pitch non-ECG-gated CT image, and ECG-gated CTA image highlighting improved visualization of heart and proximal aortic structures with high-pitch exam even without ECG gating. Abbreviations: LA, left atrium; LV, left ventricle; RV, right ventricle; S, sinus of Valsalva; DA, descending thoracic aorta

While this review is focused on detection of aortic root and proximal aortic pathologies on non-ECG-gated CT, the knowledge of CT imaging dedicated for thoracic aortic evaluation is important. CTA is the most commonly used modality for aortic evaluation. The accuracy and hence use of CTA for other vascular applications like coronary artery imaging is also expanding [11]. CT is fast, non-invasive, widely available, and allows evaluation of entire aorta with very high (nearly 100%) sensitivity and specificity for the diagnosis of aortic pathologies [2, 4, 12]. CT imaging of the proximal aorta requires appropriate timing for peak aortic contrast enhancement and conventionally needs ECG gating to prevent artifacts from transmission of cardiac pulsations. ECG-gated CTA is still considered as the standard of care for follow-up and management decisions of the aortic root and ascending aortic pathologies [13, 14]. The comparison of image quality of the heart and proximal aorta on a routine non-ECG-gated exam, high-pitch non-ECG-gated exam, and ECG-gated CTA exam is shown in Fig. 1. ECG gating can be performed prospectively or retrospectively. In prospective ECG triggering, the tube current is switched on (equivalent to image acquisition) only during a specific phase (typically diastole) of the cardiac cycle with significantly reduced radiation dose as compared to retrospective ECG triggering whereby multiphase data is acquired through the cardiac cycle, and the desired phases are selected for reconstruction afterward. Retrospective triggering leads to higher radiation dose. The use of ECG-based tube current (mA) modulation significantly decreases the dose in retrospectively triggered scans. The dedicated CT protocol for aortic pathologies is summarized in Table 1.

**Table 1:** Dedicated CT angiography protocol for thoracic aortic pathology

Thoracic aorta CTA protocol at our institute	
Scan range	Full chest coverage from the thoracic outlet through the lung base
Contrast phases	1. Non-contrast high-pitch non-ECG-gated chest (pitch factor > 3) 2. Contrast-enhanced ECG-gated chest
ECG gating	Prospective with automatic single-phase selection based on the HR (75% for low HR and 40% for high HR)
Radiation exposure	kV automatically adjusted from the topogram otherwise adjusted based on patient size 120 (BMI > 30), 100 (BMI 20–30), 80 (BMI < 20) mA automatically adjusted from the topogram
Contrast	Iopamidol 370 mg/mL (Bracco Diagnostic, Princeton, NJ, USA)
Contrast dose	100 mL
Injection rate	4 cc/s
Slice thickness	3.0 mm thickness with 3.0 mm interval in axial, coronal, and sagittal planes 1 mm thickness with 0.8 mm interval for 3D postprocessing and double-oblique measurements

High-pitch scanning possible at dual-source scanners

*CTA* CT angiography, *ECG* electrocardiogram

### 3D imaging and centerline analysis

When evaluating relatively tortuous segments like aortic arch, precise localization of pathology should be performed using the double oblique technique. The aortic diameters should always be assessed in the short axis to the centerline using a double oblique technique on a 3D workstation to prevent overestimation on raw axial CT images. The maximum transverse diameter of the aorta perpendicular to the centerline parallel to the wall of that segment of the aorta is measured. These techniques allow reproducible aortic measurements at specific anatomic landmarks. The maximum diameter of the SOV is measured in short axis from the sinus-to-trigon, and the ascending aorta is typically measured at the level of the right pulmonary artery [2, 15]. Since the treatment and follow-up of many aortic pathologies are based on size cutoff, a standardized methodology is very important (class A recommendation) [13]. Other 3D-techniques, including volume rendering (VR) and maximum intensity projections (MIP) images, can be reconstructed from the raw CT images. These images are supplementary to the primary CT angiography (CTA) images and are helpful for surgical planning [2, 16]. Cinematic rendering (CR), a relatively new 3D technique which allows realistic shadowing effects which enable clear representation of the relative positions of objects within the imaged volume, is also being increasingly used for aortic pre-surgical planning and differentiating pathologies from normal variants [17, 18]. As compared to VR, CR has improved ability to visualize the spatial relations (particularly in the through-plane) [18].

### Aortic anatomy—aortic root and ascending aorta

**Fig. 2:**
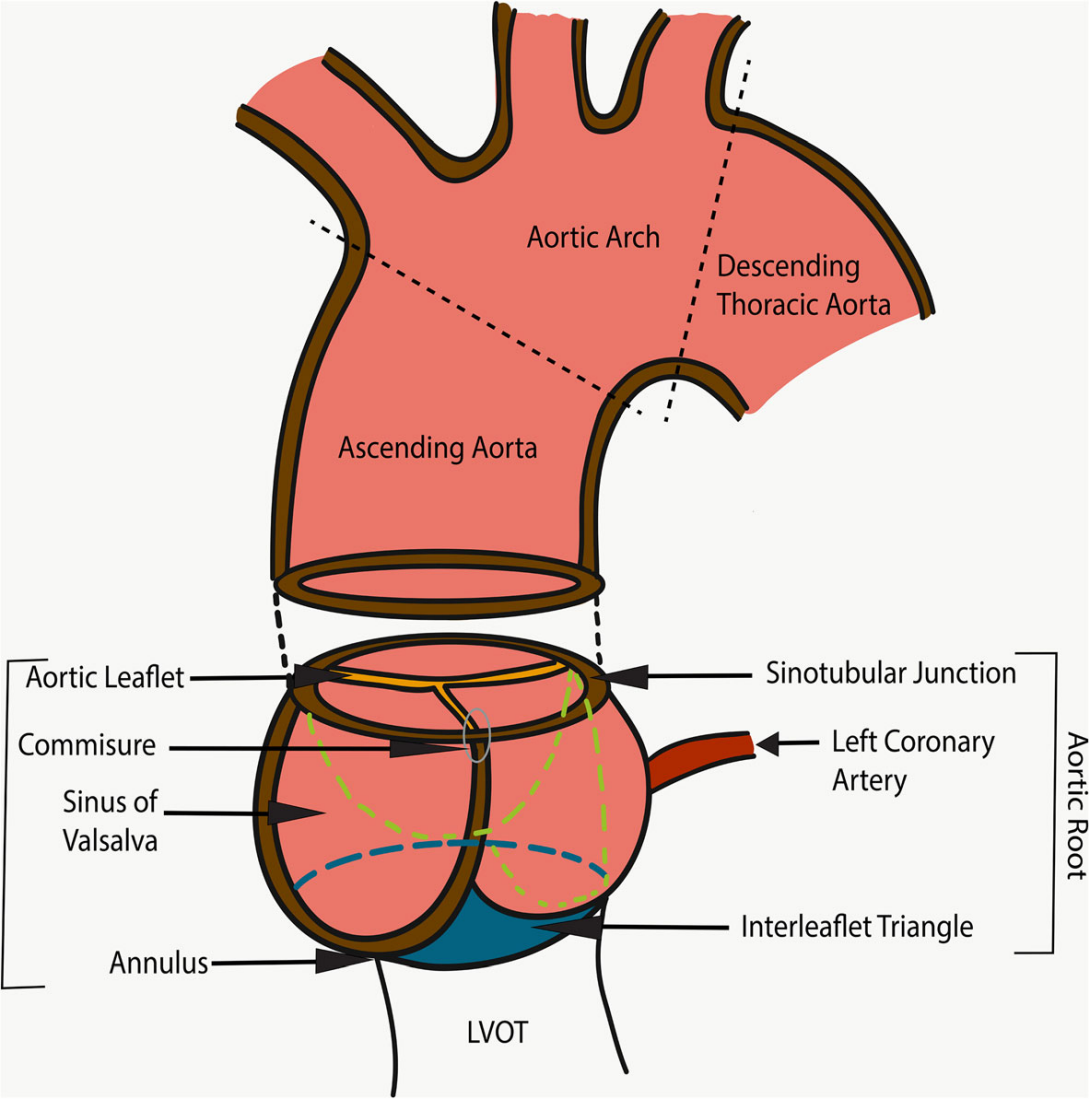
Illustration demonstrating the anatomy of the aortic root

**Fig. 3:**
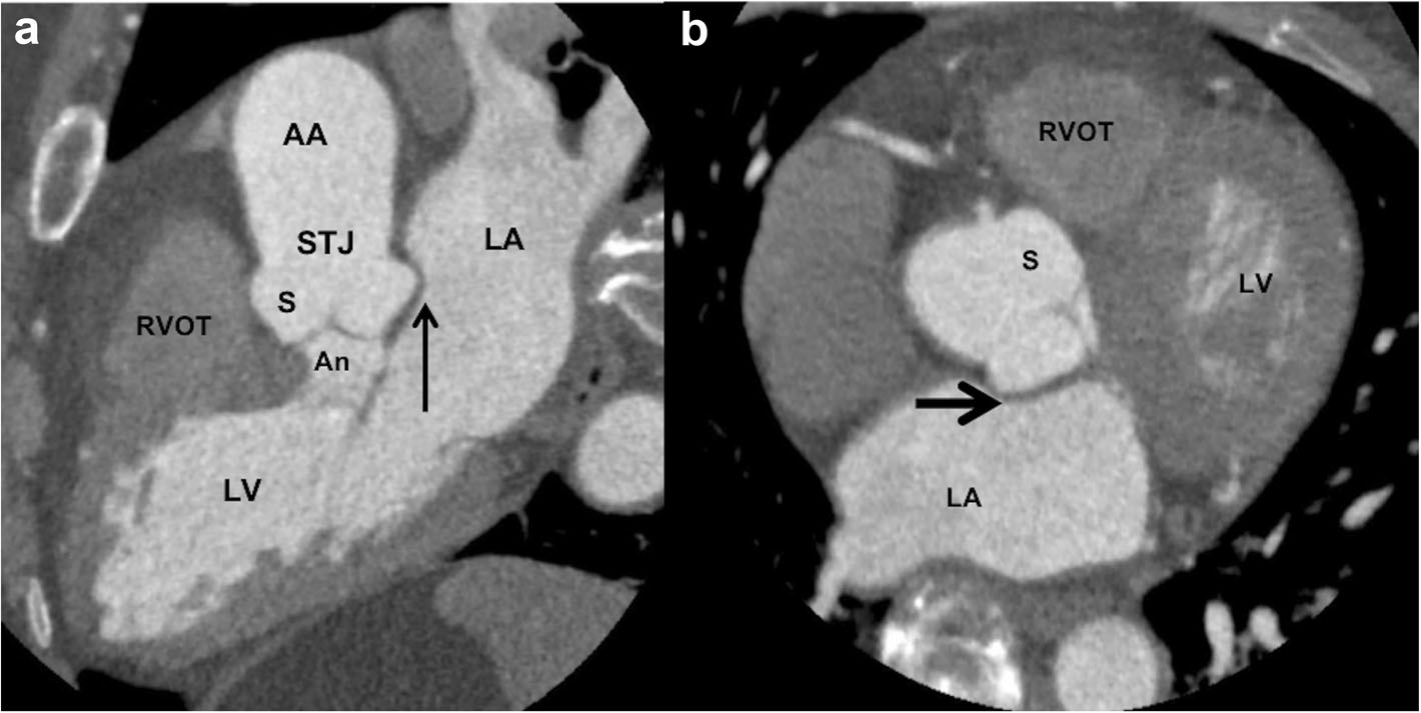
Reformatted 3-chamber view (**a**) and axial (**b**) CTA of the heart and proximal aorta showing the normal relation of the aortic root to the left atrium. No soft tissue should be present between the wall of the left coronary sinus and the left atrium (black arrow). In patients with aortic root infection, this space (black arrow) is increased with soft tissue. Abbreviations: AA, ascending aorta; LA, left atrium; LV, left ventricle; PA, pulmonary artery; An, annulus; S, sinus of Valsalva; STJ, sinotubular junction

**Fig. 4:**
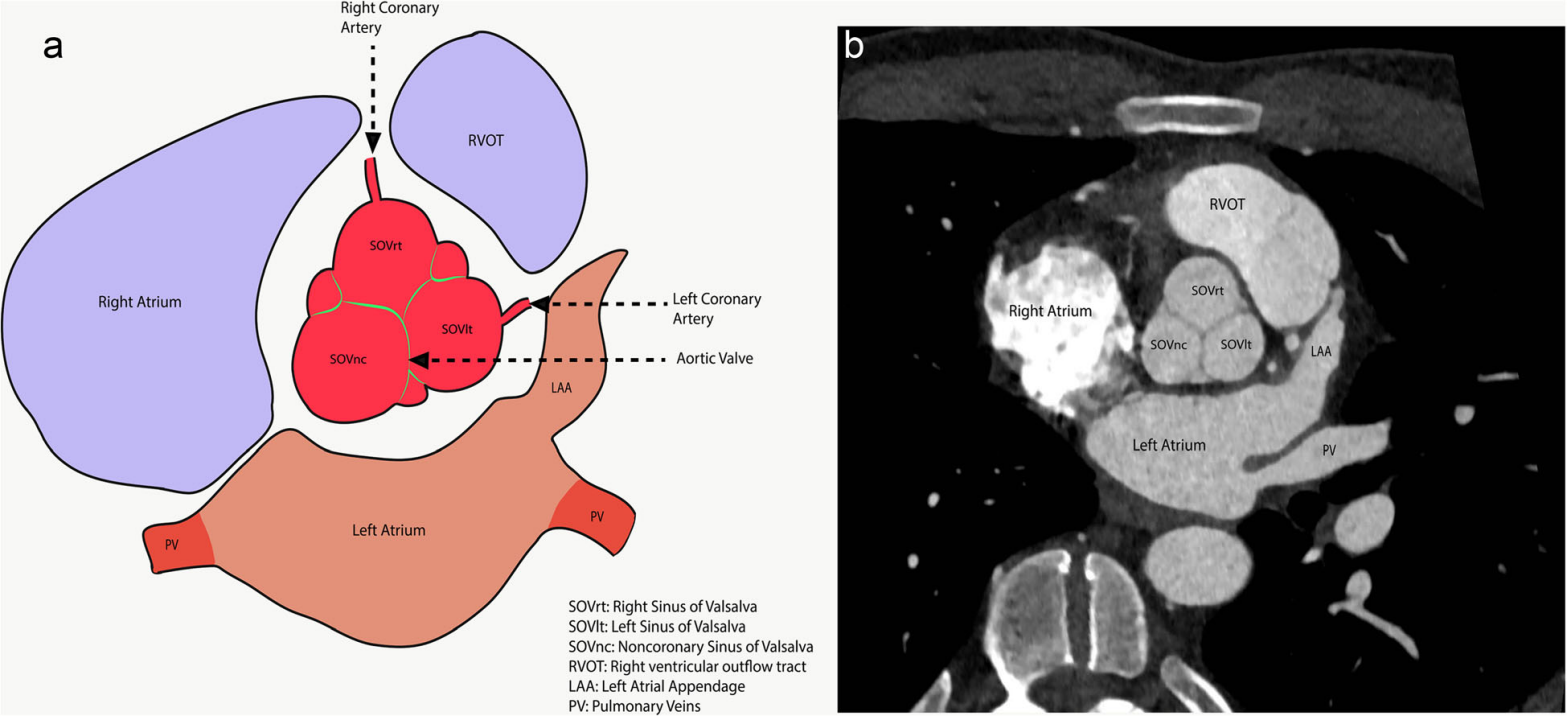
An illustration (**a**) and a CTA short axis image (**b**) through the sinuses of Valsalva highlighting the nomenclature and anatomy

The left ventricle outflow tract (LVOT) continues into the systemic circulation as thoracic aorta. The aortic root is the bridge between the LVOT and the ascending aorta. Aortic root is the proximal-most segment of the aorta from the aortic annulus to the sinotubular junction. In surgical literature, the aortic annulus is described as the plane of attachment of aortic cusps with the aortic wall [19]. In radiology and cardiology literature, the aortic annulus is described as the nadir of the attachment of the aortic leaflets. Components of the aortic root include the aortic annulus, aortic leaflets with their attachments and trigones, the sinuses of Valsalva (SOV), and the sinotubular junction (STJ) (Fig. 2). The three leaflets form the aortic valve and provide its main sealing mechanism [20]. In healthy individuals, the aortic root is directly anterior to the left atrium with no soft tissue in between (Fig. 3). SOV are the three bulges in the aortic root, between the valve and the sinotubular junction (Fig. 2). SOV are named based on the coronary origin. The anterior bulge from which the right coronary artery normally originates is the right SOV, and the left posterior bulge from which the left coronary artery normally originates is the left SOV. The right posterior bulge which faces the interatrial septum and has no coronary artery origin is called the non-coronary SOV. The illustrative and CTA anatomy of the SOV is highlighted in Fig. 4. The ascending aorta is the part of the aorta between the sinotubular junction and the origin of the first arch vessel. Normally, the proximal aorta lies posterior and to the right of the pulmonary artery.

The normal diameter of the aortic root and ascending aortic diameter is influenced by patient age, gender, and body surface area. In 3431 Framingham Heart Study participants, ECG-gated CT showed mean diameter of 34.1 ± 3.9 mm for the proximal thoracic aorta for men and 31.9 ± 3.5 mm for women [21]. Similarly, a study on SOV diameter in adults demonstrated that mean diameter in end-diastole is 3.2 ± 0.6 cm for men and 2.9 ± 0.5 cm for women [22]. Due to variations in size with patient age, gender, and body surface area, having a single diameter cutoff for abnormal diameter is frequently inaccurate. However, the traditionally accepted values for the upper limits of normal diameter for SOV and the STJ are 4 cm and 3.6 cm for males and 3.6 cm and 3.2 cm for females respectively [23, 24]. The maximum transverse aortic diameters are measured in the short axis using the double oblique method or centerline analysis at defined anatomical landmarks.

### Lesions of the proximal aorta

#### Ascending aortic and sinuses of Valsalva aneurysm

An aortic aneurysm is defined as an abnormal permanent dilatation of the aorta to greater than 1.5 times its expected normal diameter [2]. Among all thoracic aortic aneurysms, 60% comprises of the aortic root, ascending aorta, or both [25]. True aneurysms involve all the layers of vessel wall while a false aneurysm/pseudoaneurysm represent a disruption of layers of the wall of the aorta with containment of extravasated blood by surrounding tissues forming a pseudo-capsule [26]. Based on the normal variation in the size of the proximal aorta (described in “Aortic anatomy—aortic root and ascending aorta” section), the American College of Radiology (ACR) white paper on management of incidentals on thoracic CT suggest a size of 5 cm as the cutoff for a proximal aortic aneurysm [27]. When using such approach of a cutoff number, it should be kept in mind that aortic diameter is a factor of sex, age, patient size, and the segment of the aorta [13]. If the maximum aortic diameter is between the upper limits of normal (SOV, STJ: 4 cm, 3.6 cm for males and 3.6, 3.2 cm for females [23, 24]) and not meeting the criteria for aneurysm, the abnormal segment of the aorta should be reported as dilated [27]. For increased accuracy, the maximum aortic diameter can be indexed to body surface area. For proximal aorta, the value of 2.1 cm/m^2^ as an upper limit of normal and a value of 2.75 cm/m^2^ as a cutoff for aneurysm has been described to have specificity of > 95% [14, 23]. The presence of connective tissue disease can be an important determinant in diagnosis and management of an aortic aneurysm. As per American Heart Association and European Society of Cardiology guidelines [13, 14], patients with known connective tissue disease should be treated early at smaller aortic diameters because of increased risk of aortic dissection and rupture. In patients without any aortopathy, guidelines suggest a cutoff of ≥ 5.5 cm. For patients with bicuspid aortic valve or Marfan syndrome, a cutoff for treatment is between 4.5 to 5.5 cm, depending on risk factors and clinical context [2, 13, 14]. Loeys-Dietz syndrome patients either follow similar criteria as Marfan syndrome patients or are treated at a diameter ≥ 4.2 cm, given lack of consensus [2, 13, 25].

**Fig. 5:**
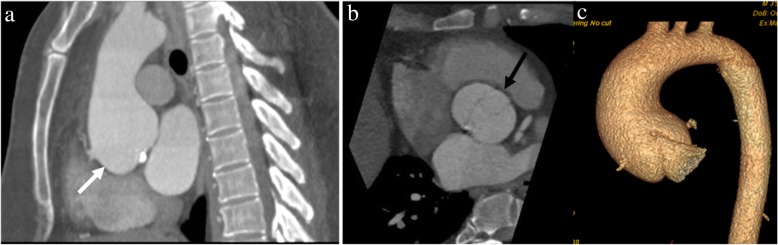
A 43-year-old man with Marfan syndrome and bicuspid aortic valve: sagittal (**a**) and axial (**b**) CTA images shows aortic root aneurysm with dilatation centered at the sinuses of Valsalva (white arrow in **a**) with effacement of the sinotubular junction and normal caliber ascending aorta and bicuspid aortic valve (black arrow in **b**). A “tulip-shaped” configuration of the aortic root is better appreciated on the volume rendered image (**c**)

**Fig. 6:**
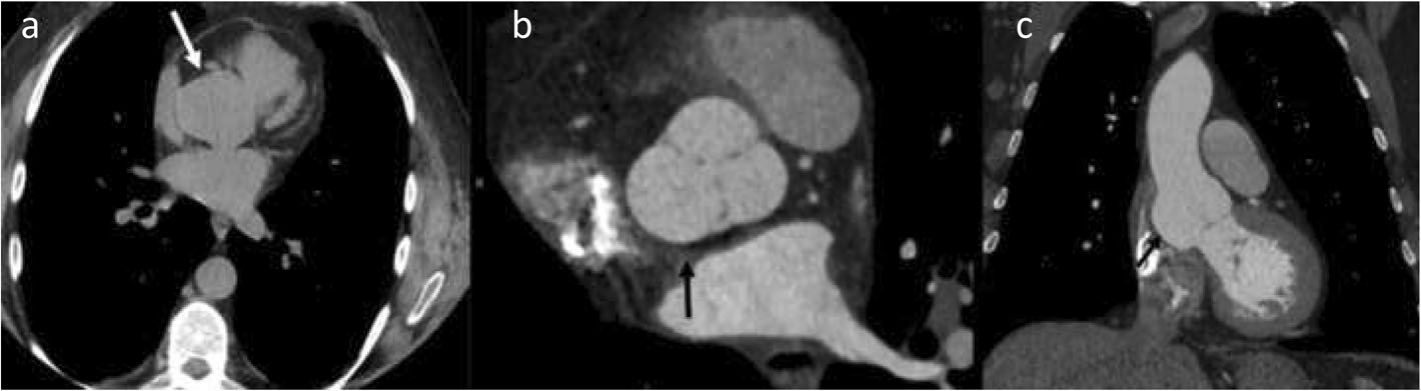
A 41-year-old-man with incidentally detected sinus of Valsalva aneurysm. Non-contrast chest CT (**a**) obtained as a work-up for fever showed an incidental dilatation (white arrow) of the aortic root (CT was done with high pitch, enabling the anatomic evaluation of aortic root despite the lack of ECG gating). Follow-up contrast-enhanced-gated CTA images (**b** and **c**) showing an incidental aneurysm of the noncoronary sinus of Valsalva (black arrow)

Within the aortic root, aneurysms may be seen isolated at the SOV or involving the entire aortic root. Combined involvement of aortic annulus, SOV, and sinotubular junction, also known as annuloaortic ectasia, is characteristic for connective tissue disorders like Marfan syndrome and Ehlers-Danlos syndrome (Fig. 5) [15]. On the other hand, isolated SOV aneurysm is mostly congenital and less commonly associated with infection, atherosclerosis, degenerative disease, or trauma [28]. Congenital SOV aneurysm is seen in approximately 0.1% of the population, is common in Asians, and is most commonly seen at the right aortic sinus followed by the noncoronary sinus (Fig. 6) [15]. SOV aneurysm can also be associated with other congenital heart diseases like ventricular septal defect (30–60%), aortic insufficiency (20–30%), bicuspid aortic valve (10%), aortic stenosis, infundibular pulmonary stenosis, patent ductus arteriosus, left ventricular non-compaction, atrial septal defect, coronary artery anomalies, and persistent left-sided superior vena cava [15]. Commonly, these aneurysms are diagnosed incidentally. However, symptoms may be related to aneurysm rupture or mass effect on the adjacent structures [28]. The rupture most commonly happens into the right ventricle and the right atrium. Other less common sites include right ventricular outflow tract, left ventricle, interventricular septum, or left atrium [15, 28]. The rupture into the pericardium is very rare but has high mortality [29]. The other sites of rupture have relatively less mortality, with mean survival after diagnosis being 3.9 years [28]. The treatment and follow-up for unruptured SOV aneurysm are frequently debated. It is suggested that unruptured SOV aneurysms should be anticoagulated and followed up every 6 months [30].

#### Pseudoaneurysm

**Fig. 7:**
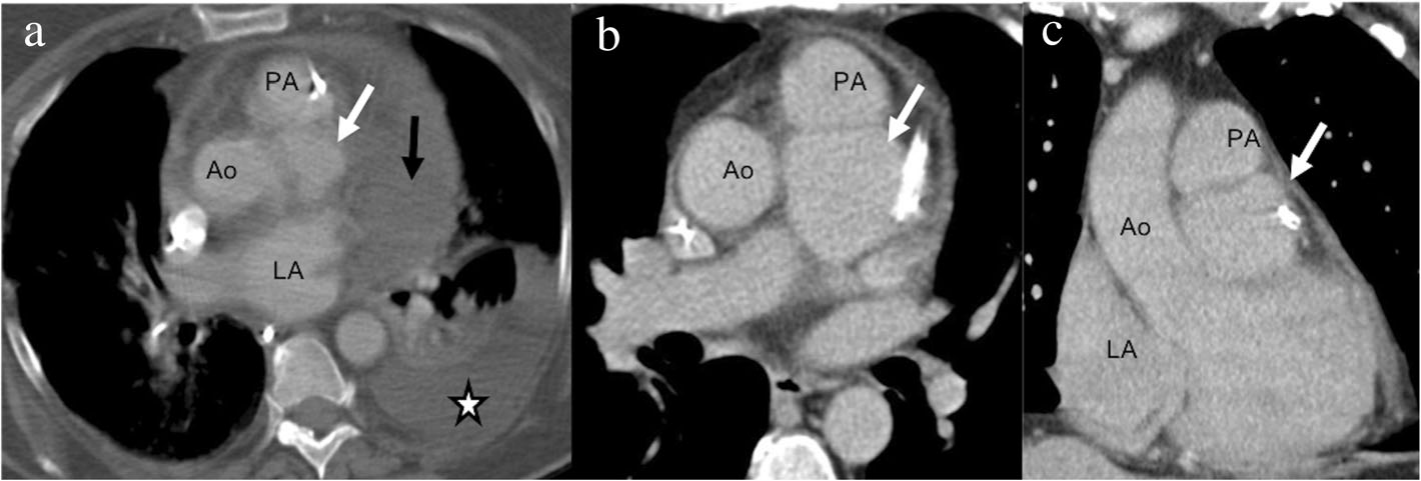
A 55-year-old female with *Staphylococcus aureus* sepsis, cardiogenic shock, and pulmonary lesions concerning for septic emboli. Axial (**a**) non-ECG-gated chest CT showed hyperdense purulent pericardial effusion (black arrow in **a**) with a contrast filled outpouching at the aortic root (white arrow) and left pleural effusion (star). The contrast outpouching was not recognized by a non-cardiovascular imager. Follow-up axial (**b**) and coronal (**c**) high-pitch CT images (venous phase) after 1 month of treatment showed resolution of pericardial effusion but enlarged pseudoaneurysm (white arrow) exerting mass effect on the left anterior descending coronary stent. Surgical repair of the pseudoaneurysm with coronary artery bypass grafting was performed due to continued chest pain

Blunt thoracic trauma (related to motor vehicle accidents, falls, and sports injuries), post-surgical, and infection are the most common cause of the pseudoaneurysms of the heart or the thoracic aorta [31]. Pseudoaneurysms can be complicated with fatal rupture, fistula formation, and compression of surrounding structures (Fig. 7). Overt free rupture can occur due to complete disruption of all of three layers of the aortic wall leading to massive hematoma with resultant hemodynamic instability. Patients with an aortic pseudoaneurysm are characterized on imaging with perivascular hematoma sealed off by periaortic structures like mediastinum, pleura, or pericardium. Non-contrast scan is helpful to identify areas of contrast enhancement and to differentiate pseudoaneurysm from calcifications and prior surgical changes. Management of aortic pseudoaneurysm involves either endovascular intervention or open surgical repair and is independent of its size [14]. Rarely, cardiac pseudoaneurysm may be managed medically with serial imaging surveillance [31].

#### Aortic dissection (AD)

An AD is characterized by an intimomedial tear of the aortic wall with subsequent separation of the layers. Dissections most commonly arise in the ascending aorta 1 cm distal to the sinotubular junction or in the descending aorta at or just beyond the isthmus of thoracic aorta because of maximum wall shear stress [32]. Spontaneous dissections that originate in the aortic root are rare. Most AD with aortic root involvement is due to retrograde dissection from the ascending aorta which increases chances of rupture into the pericardial space causing cardiac tamponade, dissect into coronary artery origin, or create aortic valvular regurgitation [33]. These complications are life-threatening and therefore warrant urgent surgical repair. The involvement of the origin of coronary arteries can lead to ischemia from extension of the dissection into the ostia or by narrowing from the intimomedial flap within the aorta without extension into the coronary artery. The right coronary artery is most commonly affected [32].

**Fig. 8:**
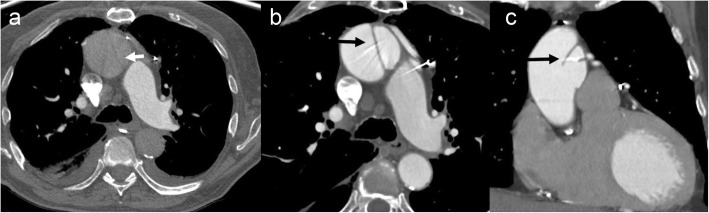
A 75-year-old man with shortness of breath and chest pain that underwent high-pitch CTA pulmonary artery (**a**) that was negative for pulmonary embolism but a concern for aortic dissection was raised by the cardiovascular radiologist. Aortic protocol CTA confirmed a type A dissection with entry point adjacent to coronary bypass graft (arrow in **b** and **c**) that was very subtle on the CTA pulmonary artery (**a**)

CTA with ECG synchronization is the standard of care for the diagnosis of AD with very high sensitivity and specificity [12, 34]. However, the recent British Society of Cardiovascular Imaging/British Society of Cardiovascular CT guidelines mention that based on scanner capabilities, motion-free CT imaging without ECG synchronization on newer scanners may be appropriate for suspected acute aortic syndrome (AAS) [35]. On non-contrast or inappropriately timed contrast-enhanced CT, diagnosis of AD may be challenging (Fig. 8a). On non-ECG-gated CT/MRI images, complications of ascending aortic dissection (such as extension of dissection into the coronaries, or rupture into pericardial space) can be missed due to motion artifact, and ECG-gated images or high-pitch CTA (if scanner is capable) should be obtained whenever there is high suspicion (Fig. 8b, c).

#### Intramural hematoma (IMH)

Aortic IMH is pathologically characterized by a hematoma in the media of the aortic wall with an absence of a well-defined enhancing false lumen. Initially, these were thought to be from rupture of vasa vasorum but increasingly small intimal ulcer like projections are being recognized which is proposed to lead to hematoma within the wall [36]. As per the analysis of the International Registry of Acute Aortic Dissection [37], the clinical presentation and prognosis of type A (aortic root and ascending aortic) IMH is similar to type A AD. However, type A IMH patients were more likely to have periaortic hematoma and pericardial effusion which are important to recognize on imaging.

**Fig. 9:**
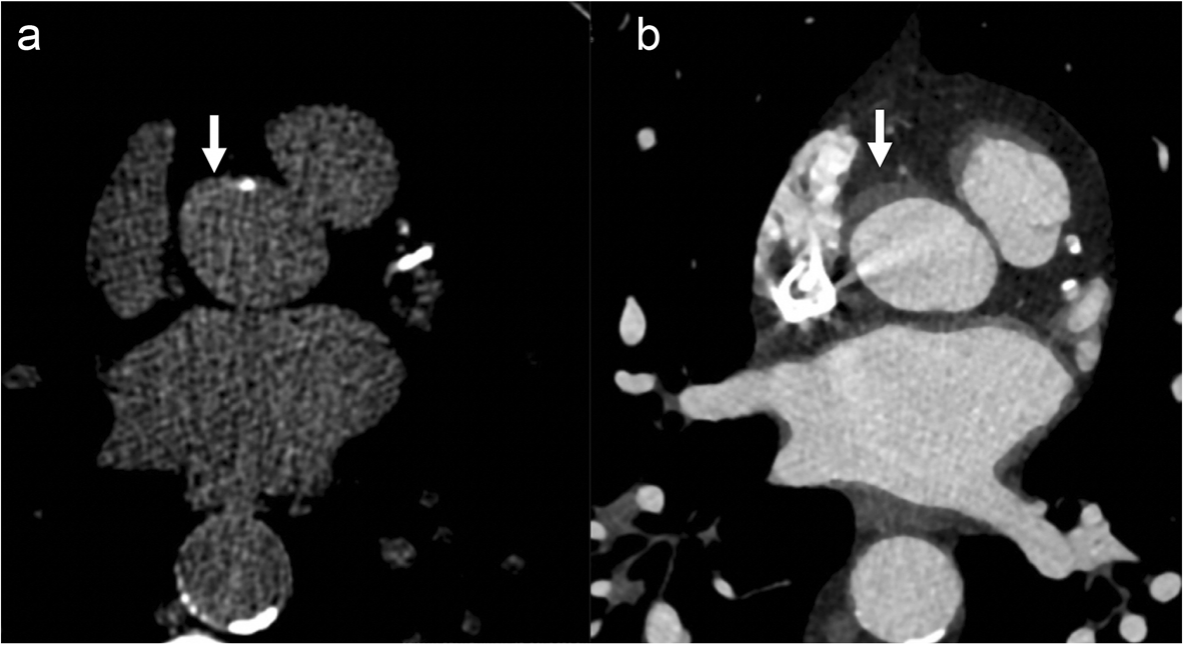
A 72-year-old male with chest pain after conventional angiography done as a part of valve-in-valve surgical clearance. Non-contrast (**a**) and contrast-enhanced (**b**) CTA images showing hyperdense aortic wall thickening at the sinotubular suggesting focal intramural hematoma, related to intimal injury from difficult right coronary artery cannulation

Non-contrast CT is very helpful in patients with IMH as high attenuation of wall hematoma is characteristic with an absence of intimal flap or enhancement on contrast-enhanced CT (Fig. 9). MRI using vessel wall imaging, black blood, and cine gradient echo sequences may be used as a problem-solving tool to differentiate IMH from atherosclerotic wall thickening and thrombus and to characterize the age of IMH [12, 36]. The complications and management of IMH is similar to AD.

#### Proximal aortic infections

**Fig. 10:**
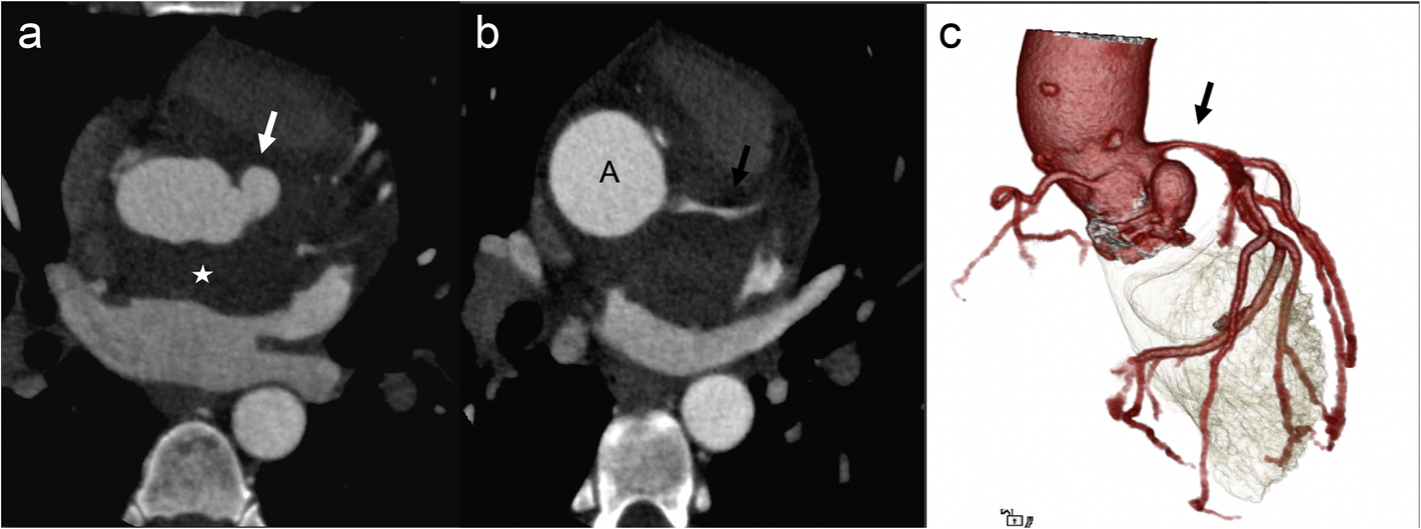
A 33-year-old male with a history of intravenous drug abuse with infectious endocarditis status post aortic valve replacement, presenting with fever. Axial (**a** and **b**) and volume-rendered (**c**) CTA images showing soft tissue (star) between the aortic root and the left atrium consistent with a paraaortic abscess. A pseudoaneurysm (white arrow) is also seen, and there is a mass effect on the left main coronary artery (black arrow) with moderate narrowing

Most commonly, proximal aortic infections are periannular and supraannular extensions from aortic valve endocarditis. Sometimes, aortic root infection may be incidentally seen in patients getting CT for fever or sepsis evaluation. Fat stranding around the proximal aorta, obliteration of pericardial and mediastinal fat, development of soft tissue attenuation at the aortic root (especially between the aortic wall and the left atrium, Fig. 10), and a frank fluid collection with enhancing rim are often the imaging features of a perivalvular extension of the infection. The complications include an extension to the mitral valve via aortomitral intervalvular fibrosa, thrombosis, or narrowing of the coronary artery (Fig. 10), fistulous connections with the adjacent cardiac chambers, or rupture [38–40]. Aortic root involvement can also be seen as wall thickening or a pseudoaneurysm.

Imaging plays an important role in the diagnosis of these infections and is very helpful for pre-surgical planning. With an improved spatial and temporal resolution of CT, even valve vegetations can sometimes be recognized [41]. Although transthoracic and transesophageal echocardiograms are often the first imaging modalities in the diagnosis of infective endocarditis, ECG-gated CTA provides a comparatively better anatomic assessment in regards to the extension of periannular abscess into adjacent cardiac valves, myocardium, coronary arteries, and pericardial space which has been highlighted in the recent studies and guidelines [42–45].

#### Aortitis

Inflammation of the aortic wall can be either infectious or non-infectious. Both infectious and non-infectious etiologies can involve proximal aorta. Among the inflammatory etiology, aortic involvement is classically seen with large vessel vasculitis most commonly giant cell arteritis (GCA) and Takayasu’s arteritis (TA). Chronic infectious causes of aortitis, including HIV, tuberculosis, and syphilis, demonstrate a non-specific imaging appearance of aneurysm formation with or without wall thickening which in the absence of other supporting imaging findings cannot be reliably distinguished based on their imaging appearance [46, 47]. Other etiologies of aortic root aortitis include radiation-induced vasculitis in patients with prior therapeutic radiation to the chest wall and are often encountered in the form of accelerated wall calcifications [48]. Within large vessel vasculitides, GCA is more likely to involve the aortic root and ascending aorta [49] while TA most commonly involves abdominal aorta followed by descending thoracic aorta and aortic arch [50, 51].

**Fig. 11:**
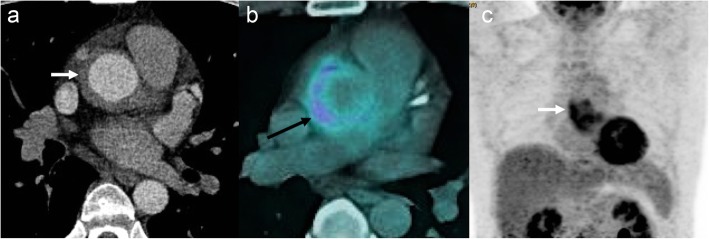
A 47-year-old male with atypical chest pain. Axial CTA image (**a**) shows circumferential wall thickening (arrow) of the ascending aorta. Axial fused and coronal maximum intensity projection (MIP) F-18 FDG PET-CT images (**b** and **c**) shows increased FDG uptake in aortic wall (arrow) consistent with active vasculitis (temporal artery biopsy showed giant cell arteritis)

On imaging, it typically manifests as wall thickening and enhancement. On CT, the wall thickening can mimic intramural hematoma especially in the absence of non-contrast images; the differentiation of which is crucial. The inflammatory wall thickening is not hyperdense (< 40 HU) on non-contrast images and reveals enhancement on post-contrast and delayed images as compared to non-enhancing hyperdense (> 50 HU) wall thickening of IMH [52]. The pattern is also important; circumferential thickening is a feature of inflammatory aortitis while incomplete crescentic thickening is a feature of IMH. If confusion persists, T1-weighted black blood double inversion recovery images before and after contrast enhancement [53] or FDG-PET (Fig. 11) can be used as a problem-solving tool [54, 55]. Aortitis with coronary involvement can present as ostia narrowing leading to their ischemia or diffuse wall thickening. On imaging, the sequelae of aortitis include calcifications, aneurysm, pseudoaneurysm, and thrombosis [56].

#### Tumors and tumor-like conditions at the aortic root

**Fig. 12:**
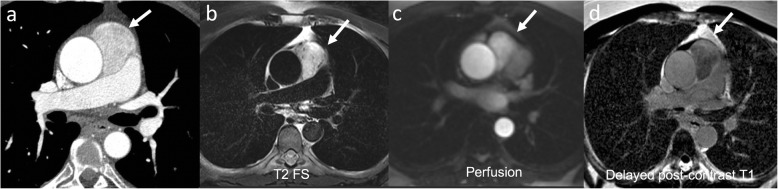
A 57-year-old male with chest pain. Axial (**a**) CTA image shows hypervascular mass between the ascending aorta and the main pulmonary artery (arrow). Further characterization with MRI (**b**–**d**) was performed, the mass had bright T2 signal with increased perfusion and wash out on delayed images. Pathology confirmed paraganglioma

Tumors and tumor-like conditions adjacent to the proximal aorta can mimic aortic root pathologies and may have overlapping clinical characteristics. Lymphomas, in particular, diffuse large B cell type, and melanoma have a predilection to pericardial space while the metastatic tumor deposits from these primary malignancies can be encountered at the aortic root in the form of enhancing mass like nodularity with associated pericardial effusion [57]. Mediastinal lymphoma can be seen as isodense to hyperdense thickening on CT with homogenous modest enhancement on post-contrast images [58]. Aortopulmonary window paragangliomas adjacent to the aortic root arise from the parasympathetic system and are rare tumors along the proximal aorta. They are usually benign, arterially enhancing homogenous tumors (Fig. 12). They can recruit vascular supply from coronaries arteries and maybe in close relation to the aortic root [59]. Their intense vascularity should warrant against percutaneous biopsy. These can be differentiated from true aortic root pathology based on maintenance of fat plane with aortic wall while confirmation of the diagnosis can reliably be obtained with an octreotide or ^131^I-metaiodobenzylguanidine scan [60].

**Fig. 13:**
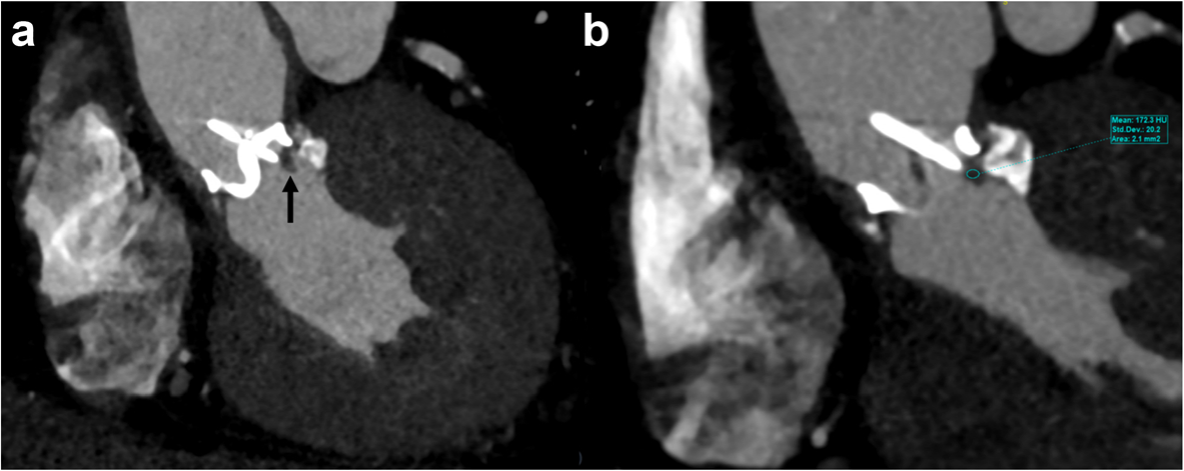
A 64-year-old male status post aortic valve replacement using Hall tilting disc valve (Medtronic, Inc., Minneapolis, Minn) and shortness of breath with increasing prosthetic aortic valve gradient on echocardiogram. Coronal oblique CTA reconstructed images reveal ovoid hypoattenuating lesion (Hounsfield unit, 172) at the inferior surface of the valve ring causing restricted valve opening, consistent with pannus formation

**Fig. 14:**
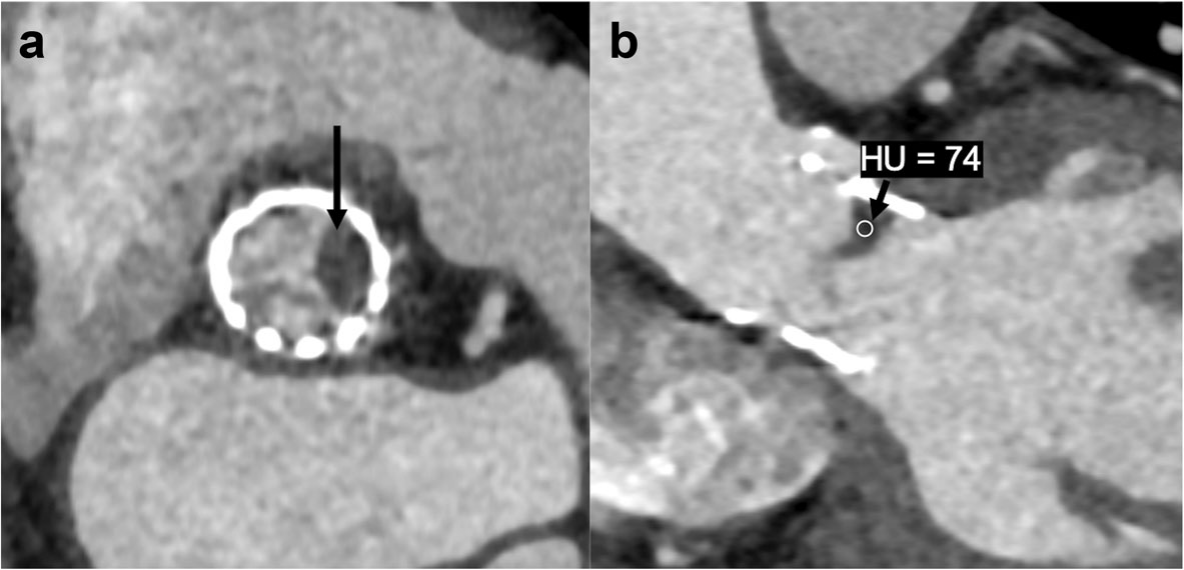
A 67-year-old female status transcatheter aortic valve replacement (TAVR) using Sapiens valve (Edwards Lifesciences) with suspected aortic valve lesion on echocardiogram. Axial (**a**) and coronal oblique (**b**) reconstructed CTA images reveal biconvex hypoattenuating (Hounsfield unit, 74) leaflet and left cusp thickening causing restricted motion (seen on cine), consistent with thrombus

**Fig. 15:**
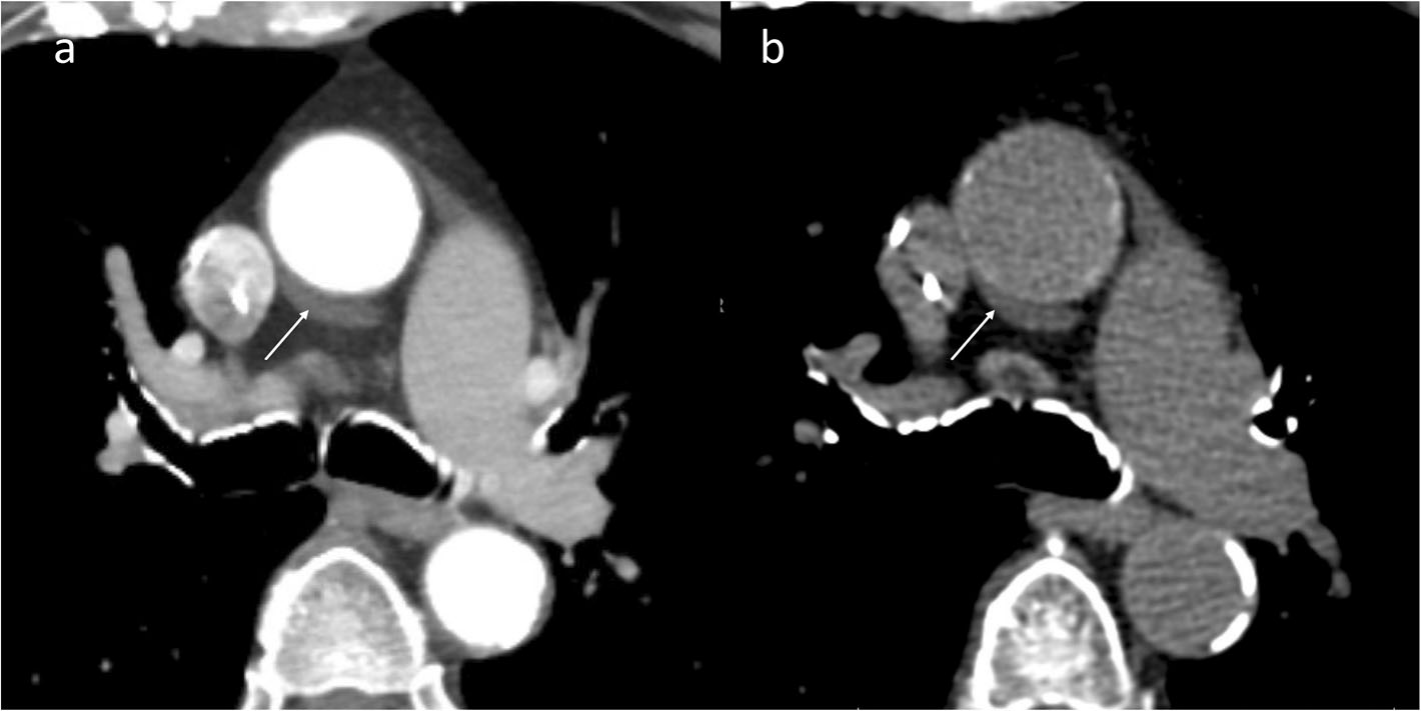
A 65-year-old male with acute chest pain. Contrast enhanced axial CTA (**a**) image shows asymmetrical hypodensity adjacent to right lateral wall of ascending aorta (arrow). Non-contrast CT (**b**) confirmed hypodense fluid attenuation consistent with superior pericardial recess; potential mimic of intramural hematoma

Non-neoplastic aortic root pathologies include aortic valve pannus of the prosthetic valve and leaflet thrombus. Pannus is a diffuse or mass-forming inflammatory process often originating from a suture [61]. The fibrotic proliferation can often occur on the ventricular side and appear as a focal round hypodensity [62]. The distinction of pannus from thrombus may be difficult but is important as both the entities have different therapeutic approaches. The pannus characteristically occurs after 12 months, is underneath the valve surface extending from the sewing ring or the metal ring, may show contrast enhancement, and has CT attenuation of > 145 Hounsfield units (HU) (Fig. 13). The thrombus, on the other hand, can occur at any time, can be above or below the aortic prosthetic aortic leaflets, does not enhance, and has an attenuation of < 145 HU (Fig. 14) [63]. Thrombus with an attenuation of < 90 HU is associated with the higher success of lysis [64]. A mimic of aortic thickening and mediastinal lymph node is the superior aortic recess. It is the most cephalad portion of the transverse pericardial sinus. Fluid attenuation on non-contrast CT images, absence of contrast enhancement, and characteristic location (Fig. 15) helps to differentiate this entity from other pathologies [65].

### Post-surgical appearance

#### Normal post-surgical appearance

A detail description of various aortic surgeries and their postoperative appearances is beyond the scope of this article and is well described in literature dedicated to this topic [66]. We discuss some expected post-surgical appearance of the proximal aorta that general radiologists may encounter and should be familiar with, as it may mimic abnormal conditions. Evaluation of post-operative thoracic aorta is typically performed with unenhanced and arterial phase CTA study. Knowledge of pertinent clinical information, including surgical notes, is important to recognize and differentiate normal from abnormal post-surgical appearances.

**Fig. 16:**
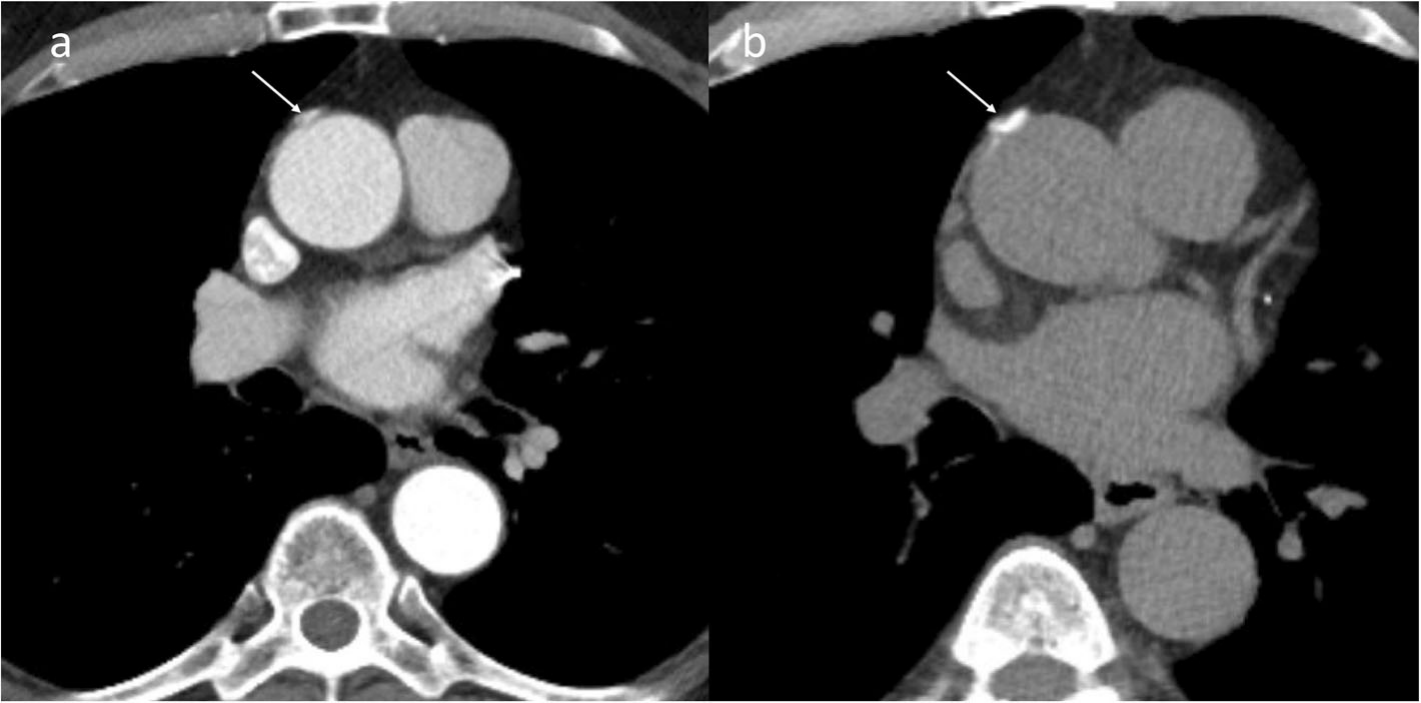
A 89-year-old female patient with acute chest pain. Contrast enhanced axial CT (**a**) image was suspicious of a small pseudoaneurysm (arrow); however, non-contrast CT (**b**) image identified the outpouching as surgical pledget (arrow)

Felt pledgets or sutures used during surgery can be mistaken for small pseudoaneurysm on a contrast-enhanced study. CT imaging can differentiate these entities as felt appears hyperdense to blood pool on unenhanced CT (Fig. 16) while pseudoaneurysm appears isodense to the blood, and felt sutures are typically symmetric at the anastomosis. If incidentally seen in a patient with chest pain, comparison with prior exam or a repeat ECG-gated CT angiography (with non-contrast images) should be obtained for differentiation as management of these entities is different.

**Fig. 17:**
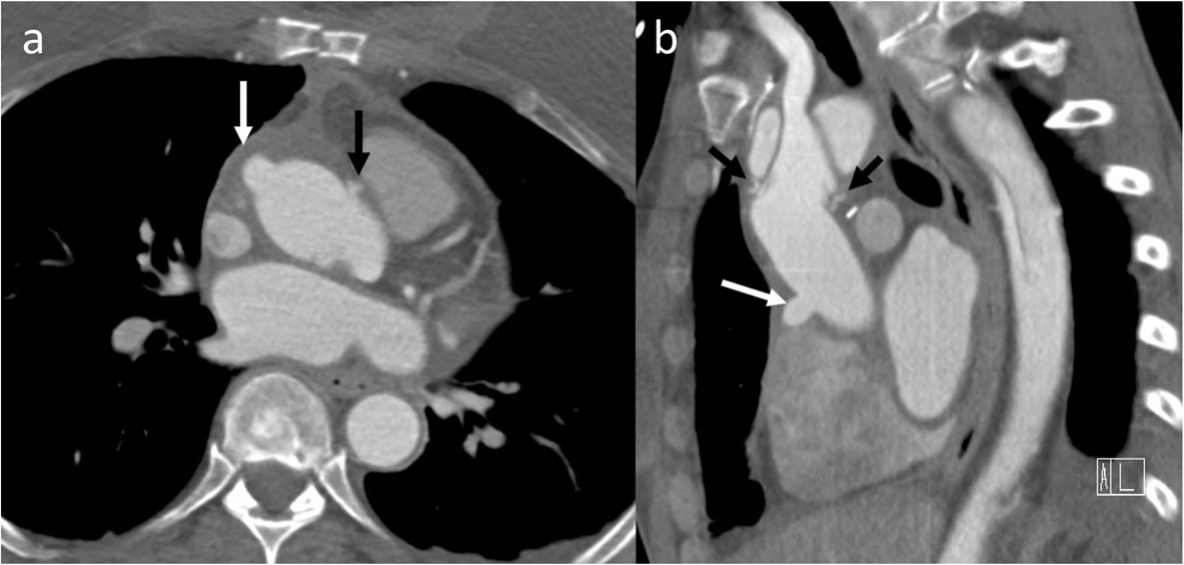
A 58-year-old female post-op day 10 status post supracoronary ascending aorta replacement with graft repair and concern of pseudoaneurysm on a pulmonary embolism rule-out CT (not shown). Axial (**a**) and sagittal (**b**) high-pitch CTA images show a smooth contrast filled outpouching from proximal graft (white arrow) near to the anastomosis (black arrow in **a**). This smooth outpouching is a normal post-operative appearance, related to over sewn reperfusion catheter stump from cardiopulmonary bypass during surgery

The cardiopulmonary bypass cannula required during various cardiothoracic surgical procedures is typically placed at the ascending aorta. An arterial perfusion cannula may also be placed through a graft side branch (if graft repair of ascending aorta is being performed) to allow antegrade systemic perfusion during the surgery till distal anastomosis is complete [67]. The bypass cannula site may appear as an outpouching at CT (Fig. 17), thereby mimicking a pseudoaneurysm or leak. Correlation with the surgical report or directly with the surgeon and correlation with patient symptoms is critical to avoid this confusion. On CT images, it appears as a well-defined, broad-based, outpouching of contrast beyond the normal contour of the aortic wall and in direct communication with the aortic lumen (Fig. 17) [60].

#### Abnormal post-surgical appearance

**Fig. 18:**
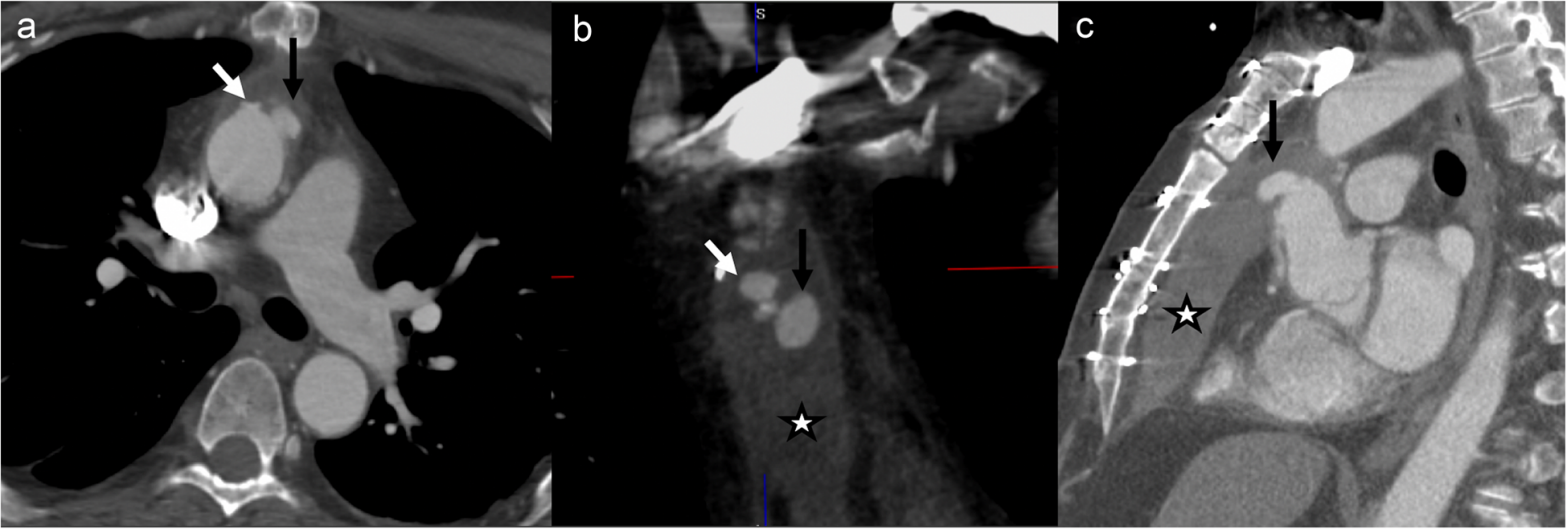
A 69-year-old male, 6-week status post open ascending aortic aneurysm repair with chest pain, presented with acute chest pain. Axial (**a**), coronal (**b**), and sagittal (**c**) CTA images show a smooth outpouching (black arrow) near graft repair consistent with perfusion catheter stump (expected post-surgical finding). Additionally, there is another irregular outpouching (white arrow), and irregularity and associated anterior pericardial hematoma (star) is concerning for a pseudoaneurysm. The patient had an emergent repeat surgical graft repair

Infections related to synthetic materials (e.g., suture) or concomitant mediastinal infection may be seen on CT imaging as the following: (a) focal saccular outpouching (pseudoaneurysm), usually with a narrow neck, that contains contrast material and arises from the aortic wall (Fig. 10); (b) periaortic soft tissue stranding or edema; and (c) periaortic gas [66, 67]. An aortic pseudoaneurysm close to the aortic cannulation site may be difficult to differentiate but is a surgical emergency with associated high operative morbidity and mortality, given the high likelihood of a coexistent infectious state. However, it is critical to differentiate pseudoaneurysm from aortic cannulation because of difference in management. Pseudoaneurysm shows irregular outline and is associated with a surrounding hematoma (Fig. 18). Also, it is uncommon to develop a pseudoaneurysm in the middle of a surgical graft as they most commonly occur at the anastomotic suture lines.

## Conclusions

Aortic root pathologies are being increasingly recognized with use of newer-generation CT scanners which allow high-pitch exams with lower contrast and radiation dose. Awareness of aortic root pathologies is essential for early recognition and initiation of life-saving management. On conventional routine chest CT exams, cardiac motion affects precise delineation of the proximal aorta; therefore, in patients with suspected aortic root pathology, ECG-gated imaging or high-pitch CTA is recommended. Currently, the use of ECG gating is the “standard of care”. Review of operative record and comparison with non-contrast scan can help to distinguish normal versus abnormal findings in post-operative patients.

## Data Availability

Not applicable

## Abbreviations

AAS: Acute aortic syndromes
AD: Aortic dissection
CT: Computed tomography
CTA: CT angiography
CR: Cinematic rendering
ECG: Electrocardiogram
HU: Hounsfield units
IMH: Intramural hematoma
LVOT: Left ventricle outflow tract
MIP: Maximum intensity projection
MRI: Magnetic resonance imaging
SOV: Sinuses of Valsalva
VR: Volume rendering
